# Markedly increased risk of postoperative bleeding complications during perioperative bridging anticoagulation in general and visceral surgery

**DOI:** 10.1186/s13741-020-00170-4

**Published:** 2020-11-23

**Authors:** J. F. Lock, L. Ungeheuer, P. Borst, J. Swol, S. Löb, E. M. Brede, D. Röder, B. Lengenfelder, K. Sauer, C.-T. Germer

**Affiliations:** 1 Department of General, Visceral, Transplantation, Vascular and Pediatric Surgery, University Hospital of Würzburg, Zentrum Operative Medizin, Oberdürrbacher Str. 6, 97080 Würzburg, Germany; 2 Department of Anesthesia and Critical Care, University Hospital of Würzburg, Würzburg, Germany; 3 Department of Medicine/Cardiology, University Hospital of Würzburg, Würzburg, Germany; 4 Central Laboratory, University Hospital of Würzburg, Würzburg, Germany

**Keywords:** Low molecular heparin, Atrial fibrillation, Postoperative bleeding, Thromboembolism, Anticoagulation, Bridging

## Abstract

**Background:**

Increasing numbers of patients receiving oral anticoagulants are undergoing elective surgery. Low molecular weight heparin (LMWH) is frequently applied as bridging therapy during perioperative interruption of anticoagulation. The aim of this study was to explore the postoperative bleeding risk of patients receiving surgery under bridging anticoagulation.

**Methods:**

We performed a monocentric retrospective two-arm matched cohort study. Patients that received perioperative bridging anticoagulation were compared to a matched control group with identical surgical procedure, age, and sex. Emergency and vascular operations were excluded. The primary endpoint was the incidence of major postoperative bleeding. Secondary endpoints were minor postoperative bleeding, thromboembolic events, length of stay, and in-hospital mortality. Multivariate analysis explored risk factors of major postoperative bleeding.

**Results:**

A total of 263 patients in each study arm were analyzed. The patient cohort included the entire field of general and visceral surgery including a large proportion of major oncological resections. Bridging anticoagulation increased the postoperative incidence of major bleeding events (8% vs. 1%; *p* < 0.001) as well as minor bleeding events (14% vs. 5%; *p* < 0.001). Thromboembolic events were equally rare in both groups (1% vs. 2%; *p* = 0.45). No effect on mortality was observed (1.5% vs. 1.9%). Independent risk factors of major postoperative bleeding were full-therapeutic dose of LMWH, renal insufficiency, and the procedure-specific bleeding risk.

**Conclusion:**

Perioperative bridging anticoagulation, especially full-therapeutic dose LMWH, markedly increases the risk of postoperative bleeding complications in general and visceral surgery. Surgeons should carefully consider the practice of routine bridging.

## Introduction

A growing incidence of cardiovascular diseases and stroke in the elderly population is leading to an increased prescription of oral anticoagulants (OACs) (Douketis 2002). The main indications for long-term treatment with anticoagulants are atrial fibrillation, heart valve prosthesis, and venous thromboembolism. Consequently, the number of patients on OAC requiring elective surgery is strongly increasing and perioperative anticoagulation management has become a daily challenge for surgeons. Full-dose therapeutic low molecular weight heparin (LMWH) has often been recommended as bridging therapy during temporary interruption of OAC (mainly warfarin) (Spandorfer et al. 1999). Bleeding risks decreased at prophylactic dose LMWH compared to intravenous unfractionated heparin (Beldi et al. 2007). However, there has yet been no evidence concerning the efficacy of bridging for prevention of perioperative thromboembolic events. Indeed, the efficacy of bridging was recently challenged by the BRIDGE trial reporting non-inferiority of foregoing bridging for prevention of periprocedural thromboembolism (Douketis et al. 2015a). In contrast, the meta-analysis of Siegal and colleagues has shown that periprocedural bridging, in particular at full-therapeutic dose LMWH, increases the risk of bleeding complications (Siegal et al. 2012). A large register analysis (ORBIT-AF) of bridging for atrial fibrillation reported an increase of both bleeding and cardiovascular events (Steinberg et al. 2015).

Interestingly, most studies have focused on procedures with relatively low bleeding risks such as pacemaker implantation, dental, or endoscopic procedures. Some of the studies have included patients undergoing general or visceral surgery but only in relatively small proportions (e.g., 5% BRIDGE trial (Douketis et al. 2015a), 4% PROSPECT trial (Dunn et al. 2007), 10% FCSA trial (Pengo et al. 2009), 13% A Forum study (Jaffer et al. 2010)).

The aim of this study was to explore the actual burden of increased postoperative bleeding complications in general and visceral surgery and to identify risk factors associated with major bleeding.

## Patients and methods

### Study design

The data for this monocentric retrospective two-arm cohort study were collected retrospectively from our hospital electronic database. A sample size calculation was performed based on the meta-analysis of Siegal and colleagues that reported an incidence of periprocedural bleeding events of 13.1% in the bridging cohort vs. 3.4% in the non-bridged control cohort (Siegal et al. 2012). Since no specific data or perioperative bleeding events in general or visceral surgery were available, we used these numbers although they seem to be a rather conservative approach potentially underestimating bleeding incidence after surgery. To substantiate a significant group difference with a 2-sided test at a level of α = 0.05, 167 patients (> 90% power) were required per group. Assuming a prevalence of 50 general or visceral surgical procedures with perioperative bridging, a 4-year period was reviewed. Thus, all consecutive cases receiving general or visceral surgery procedures during January 1, 2011, and December 31, 2014, were analyzed. Inclusion criteria for the bridging group were age ≥ 18 years, American Society of Anesthesiologists (ASA) physical status classification system < 5, and at least one of the following ICD 10 diagnosis codes: Z92.1 (oral anticoagulation), D68.4–D68.9 (thrombophilia), and I48.0–I48.3 (atrial fibrillation). No explicit search for patients after a venous thromboembolism was possible due to the lack of a specific ICD code for that diagnosis. Exclusion criteria were emergency surgery, vascular and bariatric surgery, or endoscopic procedures.

The control group contained cases without the above-described ICD codes that individually matched patients of the bridging group based on the organ-specific surgical procedure, the age (± 5 years), and the sex. The matching cases were collected from all patients receiving surgery during the same time frame. The selection of control group cases was blinded from the study endpoint parameters.

The retrospective data analysis was approved by the local institutional ethical review board. Each patient was anonymized using a study number. Only authorized individuals had access to the database.

### Study endpoints

The primary endpoint of the study was major postoperative bleeding, which was defined as an event leading to a drop of hemoglobin > 2 g/dl and (1) operative revision or radiological intervention for bleeding control, or requiring (2) the postoperative transfusion of ≥ 2 red blood cell packs as defined by the International Society on Thrombosis and Haemostasis (Schulman et al. 2010).

Secondary endpoints were minor bleeding, thromboembolic events, length of stay, and in-hospital mortality. Minor postoperative bleeding events were defined as clinically apparent events that were documented in the patient records, e.g., hematomas or blood loss via drainages leading to a clinical significant decrease of hemoglobin concentration. Radiological incidental findings without clinical symptoms were not appraised as a bleeding event. Finally, intraoperative bleeding episodes and blood transfusions directly related to the surgical procedure were not analyzed. The analysis of intraoperative blood loss was omitted due to the lack of a reliable intraoperative documentation throughout all patients. Thromboembolic events were defined as postoperative diagnosis of stroke, transient ischemic attack, arterial embolism, cardiac infarction, deep vein, or pulmonary embolism.

### Perioperative management of anticoagulation

Perioperative bridging was performed according to an internal bridging guideline recommending full-therapeutic dose enoxaparin (1 mg/kg bodyweight b.i.d. SC) for patients with moderate or high risk of thromboembolism. The internal guideline defined all patients with atrial fibrillation as moderate or high risk of thromboembolism. An interruption of full-therapeutic dose enoxaparin 24 h preoperatively and a continuation 24 h postoperatively were recommended. Half-therapeutic dose of enoxaparin (1 mg/kg bodyweight q.d. SC) was not explicitly recommended by the guideline. Normal prophylactic dose of enoxaparin was 40 mg q.d. SC.

### Statistical analysis

Descriptive analyses are reported as the mean and standard deviation or the median and range, unless otherwise noted. Simple and multiple logistic regression analysis and odds ratios were used to identify factors associated with major postoperative bleeding. The following variables were applied for multivariate analysis: procedure-specific risk of bleeding, HAS-BLED score, malignant tumor, age > 65 years, arterial hypertension, congestive heart failure, renal insufficiency (GFR < 60), hemoglobin level, international normalized ratio (INR), platelet count, perioperative bridging, full-therapeutic dose of low molecular heparin, preoperative interruption of LMWH ≥ 24 h, and postoperative interruption of LMWH ≥ 24 h. Stepwise variable selection was applied. The level of significance was 0.05 (two-sided). IBM SPSS Statistics, version 23 (International Business Machines Corporation, Armonk, NY), was used to perform the analysis.

The risk of thromboembolism was categorized for the statistical analysis according to the guidelines of the German Society of Cardiology (Hoffmeister et al. 2010) and the American College of Chest Physicians Evidence-Based Clinical Practice Guidelines on perioperative management of antithrombotic therapy (Douketis et al. 2012). The CHADS_2_ score, validated by Gage et al. (Gage et al. 2001), was used to estimate the risk of stroke in atrial fibrillation (low risk of thromboembolism: CHADS_2_ score 0–2, venous thrombosis > 12 months, mechanical aortic valve without risk factors; moderate risk: CHADS_2_ score 3–4, venous thrombosis > 3 months, mechanical aortic valve with risk factors or biological aortic valve; high risk: CHADS_2_ score 5–6, venous thrombosis or cerebral ischemia ≤ 3 months, relevant thrombophilia, mitral valve replacement).

The procedure-specific risk of bleeding was stratified in five categories (Jaffer et al. 2010) (e.g., minimal risk: skin incision/biopsy; mild risk: ileostoma reversal, inguinal hernia, hemithyroidectomy, laparoscopic cholecystectomy; moderate risk: hemicolectomy, large incisional hernia, open cholecystectomy, thyroidectomy, gastric wedge resection; major risk: rectum resection, hemihepatectomy, gastrectomy; critical risk: extended hemihepatectomy, extended gastrectomy, pancreas head resection).

## Results

Two hundred sixty-three patients with full dataset could be included into the bridging group. The patients were predominantly male and, on average, 71 ± 10 years old. OAC indications included mostly atrial fibrillation (82%), followed by past thrombotic events and heart valve replacement. The risk of thromboembolism was low in most patients of the bridging group; only 20% yielded a high risk. The patients were anticoagulated by warfarin (76%), direct oral anticoagulants (DOACs) (9%), or only platelet aggregation inhibitors (15%).

For all patients, a single matched patient could be identified and was assigned to the control group. Thus, bridging and control groups were identical in terms of age, sex, body mass index (BMI), the surgical procedure, and its procedure-specific risk of bleeding (Table 1).

**Table 1:** Patient characteristics and preoperative risk factors

		Bridging group	Control group	*p* value^1^
*n*		263	263	
Age [years]		71 ± 10	71 ± 10	0.63
Sex [male, %]		63.5%	62.4%	0.86
Body mass index [kg/m^2^]		27 ± 5	27 ± 11	0.89
ASA physical status classification system > 2 [*n*, %]		207 (79%)	117 (44%)	< 0.001
Bridging indications	Atrial fibrillation [*n*, %]	217 (82%)	–	
	Past thromboembolic events [*n*, %]	31 (12%)	–	
	Heart valve replacement [*n*, %]	22 (9%)	–	
	Thrombophilia [*n*, %]	9 (3%)	–	
Risk of thromboembolism	Low [*n*, %]	136 (52%)	100%	< 0.001
	Moderate [*n*, %]	75 (28%)	–	
	High [*n*, %]	52 (20%)	–	
Risk of bleeding	HAS-BLED score	2.6 ± 1.0	2.2 ± 1.2	< 0.001
	High bleeding risk (HAS-BLED score > 2) [*n*, %]	46 (18%)	31 (12%)	0.11
Procedure-specific risk of bleeding	Minimal (category 1) [*n*, %]	0	0	0.62
	Mild (category 2) [*n*, %]	121 (46%)	116 (44%)	
	Moderate (category 3) [*n*, %]	90 (34%)	101 (38%)	
	Major (category 4) [*n*, %]	40 (15%)	32 (12%)	
	Critical (category 5) [*n*, %]	12 (5%)	14 (5%)	
Cardiovascular risk factors	Hypertension: blood pressure consistently above 140/90 mmHg or treated with hypertension medication [*n*, %]	219 (83%)	162 (62%)	< 0.001
	Congestive heart failure [*n*, %]	88 (33%)	18 (7%)	< 0.001
	Coronary heart disease/arterial occlusive disease [*n*, %]	89 (34%)	44 (17%)	< 0.001
	Chronic renal insufficiency ≥ stage III (GFR < 60 ml/min) [*n*, %]	97 (37%)	36 (14%)	< 0.001
	Diabetes mellitus [*n*, %]	79 (30%)	47 (18%)	0.001

^1^*p* values of continuous outcomes were calculated by a two-sided two-sample *t* test assuming equal variances; *p* values of categorical outcomes were calculated by a two-sided *χ*^2^ test or, in case of categories with less than 5 subjects, by Fisher’s exact test

Bridging group patients revealed a statistically significant different pattern of comorbidities in comparison to the control group. Most patients in the bridging group were ASA 3, whereas the control group consisted mostly of ASA 2 patients. While arterial hypertension was common in both groups, the incidence of coronary heart disease, chronic renal insufficiency, and diabetes was doubled in the bridging group. Moreover, the incidence of heart failure was five times higher.

The analyzed surgical procedures were predominantly colorectal and hernia surgery, but contained also gastric, hepatic, biliary, pancreatic, and endocrine procedures (Table 2). Approximately half of patients underwent procedures with mild risk of bleeding, whereas the other half underwent procedures with moderate and major risk of bleeding. Five percent of patients underwent procedures with critical bleeding risk in both groups.

**Table 2:** Surgical procedures and specific bleeding risk by organ

	*n*	Procedure-specific risk of bleeding^1^				Major postoperative bleeding	
		Mild (category 2)	Moderate (category 3)	Major (category 4)	Critical (category 5)	Bridging group	Control group
Surgery by organ							
Colorectal	163 (31%)	34%	45%	20%	1%	8 (9.8%)	1 (1.2%)
Hernia	102 (19%)	96%	4%	–	–	4 (7.2%)	0
Gallbladder	64 (12%)	84%	16%	–	–	2 (6.3%)	1 (3.1%)
Thyroid	54 (10%)	30%	70%			0	0
Lymphatic	43 (8%)	19%	81%	–	–	1 (5.3%)	0
Liver	32 (6%)	3%	6%	72%	19%	1 (6.3%)	0
Stomach	30 (6%)	3%	37%	43%	17%	2 (6.3%)	0
Other abdomen (e.g., adrenal gland)	24 (5%)	13%	75%	8%	4%	2 (14.3%)	0
Pancreas	14 (3%)	–	–	7%	93%	2 (14.3%)	0
Major postoperative bleeding							
Bridging group		5 (4.1%)	8 (8.9%)	7 (17.5%)	2 (16.7%)		
Control group		0	1 (1%)	1 (3.1%)	0		

^1^Percentages for each organ

Perioperative bridging was performed using full-therapeutic dose of enoxaparin in 189 patients (72%), half-therapeutic dose in 26 patients (10%), and prophylactic dose in 48 patients (18%). Preoperative LMWH was interrupted < 24 h in 59% and ≥ 24 h in 41% of patients. Postoperative administration of LMWH was continued < 24 in 64% and ≥ 24 h in 36% of patients (also in patients on full-therapeutic dose).

Postoperative bleeding complications occurred significantly more frequently in the bridging group (overall 22.1% vs. 6.1%; *p* < 0.0001). Interestingly, the increase of major postoperative bleeding events even exceeded the increase of minor bleeding events (Table 3). Accordingly, the rate of patients requiring blood transfusions and/or reoperation was strongly increased in the bridging group. These complications led to a higher incidence of reinterventions and organ failure, resulting in increased need of intensive care and prolonged length of stay. However, no increase of in-hospital mortality was observed.

**Table 3:** Postoperative complications and patient outcome

		Bridging group	Control group	*p* value^1^
Thromboembolic events [*n*, %]		2 (0.8%)	5 (1.9%)	0.45
Postoperative bleeding^2^	Major [*n*, %]	22 (8.4%)	2 (0.8%)	< 0.0001
	Minor [*n*, %]	36 (13.7%)	14 (5.3%)	0.001
Red blood cell transfusion [*n*, %]		23 (8.7%)	2 (0.8%)	< 0.0001
Reoperation [*n*, %]		40 (15.2%)	14 (5.4%)	< 0.0001
Classification of complications^3^	Grade IIIa–V [*n*, %]	61 (23.2%)	33 (12.5%)	0.001
	Grade IVa–V [*n*, %]	18 (6.8%)	8 (3%)	0.044
In-hospital mortality [*n*, %]		4 (1.5%)	5 (1.9%)	1.0
Intermediate care/intensive care [*n*, %]		118 (44.9%)	74 (28.1%)	< 0.0001
Length of stay [days]		12 ± 11	9 ± 9	0.009

^1^*p* values of continuous outcomes were calculated by a two-sided two-sample *t* test assuming equal variances; *p* values of categorical outcomes were calculated by a two-sided *χ*^2^ test or, in case of categories with less than 5 subjects, by Fisher’s exact test

^2^According to the International Society on Thrombosis and Haemostasis (Schulman et al. 2010)

^3^According to Clavien-Dindo (Dindo et al. 2004)

The procedure-specific bleeding risk had no influence on the incidence of major bleeding complications in the control group. In contrast, its risk increased from 4% in mild bleeding risk procedures to 17% in major and critical bleeding risk procedure in the bridging group (Table 2). Furthermore, the increase of major bleeding complications in the bridging group was recorded throughout all operated organs beside thyroid gland surgery.

No statistically significant difference concerning the incidence of postoperative thromboembolic events was observed between both groups. The incidence of thromboembolic events increased by the calculated risk of thromboembolism (1.0%/*n* = 4 at low risk; 1.1%/*n* = 1 at moderate risk; 3.5%/*n* = 2 at high risk; *p* = 0.31).

Univariate analysis yielded the procedure-specific risk of bleeding, arterial hypertension, perioperative bridging, full-therapeutic dose of LMWH, and pre- and postoperative interruption of LMWH as risk factors of major postoperative bleeding complications. Multivariate analysis yielded full-therapeutic dose of LMWH as the major independent risk factor of major bleeding (Table 4). In addition, renal insufficiency, the procedure-specific risk of bleeding, and the postoperative interruption of LMWH were independent risk factors. In contrast, the multivariate analysis of overall postoperative bleeding complications revealed only full-therapeutic dose of LMWH as the independent risk factor.

**Table 4:** Risk factors of major postoperative bleeding

	*p* value univariate	*p* value multivariate	Odds ratio (95% CI)
Procedure-specific risk of bleeding	0.003	0.008	1.8 (1.2–2.9)
HAS-BLED score	0.31		
Malignant tumor	0.52		
Age > 65 years	0.084		
Arterial hypertension	0.041		
Congestive heart failure	0.039		
Renal insufficiency (GFR < 60)	< 0.0001	< 0.0001	5.8 (2.3–14.6)
Hemoglobin level	0.82		
International normalized ratio (INR)	0.072		
Platelet count	0.77		
Perioperative bridging	< 0.0001		
Full-therapeutic dose of LMWH	< 0.0001	< 0.0001	18.7 (3.6–95)
Preoperative interruption of LMWH ≤ 24 h	0.007		
Postoperative interruption of LMWH ≤ 24 h	0.006	0.068	1.5 (0.9–2.2)

## Discussion

This study points out the burden of perioperative bridging OAC especially using full-therapeutic dose LMWH in general and visceral surgery. We observed a markedly increased incidence of major and minor postoperative bleeding complications throughout all surgical procedures. Bleeding events frequently required transfusions, reinterventions, or reoperations, as well as intensive care, thus leading to a prolonged length of stay. However, the postoperative bleeding events were managed successfully without effect on postoperative mortality in contrast to previous studies (Beldi et al. 2007). The descriptive analysis revealed that patients receiving OAC have frequently additional comorbidities in comparison to control patients of the same age. In particular, the incidence of renal insufficiency was more than two times higher which is an additional risk factor for postoperative bleeding complications during LMWH bridging.

The increased risk of bleeding complications and consequent reoperations for full-therapeutic dose LMWH was previously shown for arthroscopy (Gibon et al. 2014). In addition, the role of full-therapeutic dose LMWH as the major factor increasing bleeding complications was previously reported from a prospective observational multicenter trial on different procedures (Jaffer et al. 2010). Interestingly, the adjusted odds ratio of major bleeding for full-therapeutic dose LMWH was 4.4 in contrast to 18.7 in our population. This might be explained by the fact that our patient population mostly underwent procedures with a relevant bleeding risk whereas the study of Jaffer et al. included minor procedures in 63% of patients. Furthermore, a retrospective Japanese study on patients receiving major abdominal malignancy surgery demonstrated the safety of prophylactic dose heparin for bridging of OAC (Ono et al. 2016). Therefore, full-therapeutic dose LMWH should be restricted to patients with a high risk of thromboembolism.

In addition, the preoperative interruption and postoperative continuation of LMWH were not correctly performed in a relevant number of patients in this study. LMWH administration in full-therapeutic dose (if it is really necessary) should be interrupted at least 24 h prior surgery, and the postoperative continuation should be delayed for 48–72 h to ensure adequate hemostasis (Patel and Arya 2014). As an alternative (especially for medium thromboembolic risk patients), a prospective cohort study has shown the safety of a less aggressive strategy using half-therapeutic dose LMWH (Dunn et al. 2007).

Recent studies have described an increased bleeding risk with no change in the risk of thromboembolic events in the bridging and non-bridging groups (Douketis et al. 2015a; Siegal et al. 2012; Steinberg et al. 2015). These results coincide with those of our study. This study is important because it presents the results from a large cohort of patients who underwent major surgical procedures, which has not previously been investigated. The analyzed patient population includes various indications for OAC and all individual risks of thromboembolism. The analyzed surgical procedures represent typical pattern of a tertiary care hospital in Germany. The multivariate analysis proved that full-therapeutic dose of LMWH bridging was the main factor influencing bleeding complications despite other comorbidities between the bridging and the control group.

Most previous studies raised questions about the safety of bridging but did not specify the varying criteria for major bleeding. In our study, hypertension was found to be the most frequent risk factor for bleeding complications, occurring in 80% of the bridging group. Because the number of thromboembolic events was very small in the RE-LY analysis, it raises questions about the benefit of bridging, considering its risks (Douketis et al. 2015b). The recent data suggest the need for an important rebalancing of risks and benefits in preoperative bridging management (Garwood et al. 2017). Appropriate risk stratification should be used to support decision-making, considering that a chronic renal insufficiency ≥ stage III was also a significant predictor of postoperative bleeding and could affect the bridging strategy. In addition, among the factors related to severe bleeding, such as renal insufficiency, the administration of LMWH in therapeutic doses and the degree of severity of the surgical procedure require consideration.

It is worth noting that most patients on OAC only suffered from a relatively low risk of thromboembolism. The BRIDGE trial has clearly shown no benefit concerning the risk of thromboembolism for this group of patients (Douketis et al. 2015a). Moreover, a German registry has shown that the vast majority of patients with a very low risk of thromboembolism (e.g., atrial fibrillation patients with CHADS_2_-VASc-Score 0–1) actually receive OAC although this is not strongly recommended (Steffel et al. 2015).

Nevertheless, several limitations of this study require notice. The majority of patients in the bridging group received warfarin due to atrial fibrillation. Other indications and medications were less frequent and did not allow valid subgroup analysis due to small sample size. In addition, the potential benefits of bridging in patients with an individually high risk of thromboembolism could not be compared with the negative effects concerning bleeding complications. This would require a much higher number of patients and could only be provided by large multicenter studies or registries. Therefore, the conclusions of this study are mainly restricted to patients with atrial fibrillation.

No difference between OAC using warfarin and DOACs was made during this analysis. In our recent clinical practice, bridging was also performed in patients on DOACs despite the manufacturer’s recommendations. This could be explained by the fact that surgeons are frequently afraid to administer DOACs early after major surgical procedures due to the lack of evidence and potential issues with enteral resorption in the early phase after gastrointestinal surgery. Nevertheless, the RE-LY study has shown similar rates of postoperative bleeding complications for dabigatran in comparison to warfarin bridging (Healey et al. 2012).

Furthermore, significant differences in comorbidity were observed between the two study groups. The primary difference between the control group and bridging group was the indication for OAC. These indications, in particular atrial fibrillation and heart valve replacement, are likely to be associated with further comorbidities, in particular cardiovascular and renal disease. Only the minority of patients suffered from deep vein thrombosis or thrombophilia. Therefore, it seems to be consistent that the prevalence of comorbidities had to be different between the control and bridging groups. However, some of these comorbidities might influence individual bleeding risk. Thus, we calculated HAS-BLED score for both study groups and did not record a clinically relevant difference or a higher proportion of patients at high bleeding risk. Taken together, the main factor influencing postoperative bleeding events was full-therapeutic dose LMWH. The differences in length of stay and intermediate-care/intensive-care utilization have to be interpreted with caution as they might be strongly influenced by comorbidity and higher ASA scores.

In addition, the locally applied bridging guideline was rather aggressive by recommending full-therapeutic dose LMWH for all patients with atrial fibrillation. This resulted in a very high proportion of patients at major bleeding risk. Clinics with other, less aggressive bridging standards are likely to see a lower incidence of bridging-associated bleeding events. Finally, no analysis of intraoperative blood loss was applicable and the definition of minor postoperative bleeding complications remains somehow weak. Therefore, the conclusions of this study are limited on major postoperative bleeding complications.

However, the results of this study are specifically important for perioperative clinicians, who face an aging population with an increased prevalence of coronary disease and atrial fibrillation leading to a higher stroke incidence and more frequent use of OAC. All reported procedures could not be performed without discontinuation of OAC. However, most clinicians do not yet seem to be focused on the issues related to bridging OAC and individual risk-adjusted bridging strategies have not yet spread throughout surgical practice (Lock et al. 2018).

OAC for patients with atrial fibrillation is a secondary prevention of thromboembolism, in particular stroke, reducing its risk about 60% (Hart et al. 2007). However, the individual risk of thromboembolism is relatively low with 2–4% p.a. in two third of patients (Agarwal et al. 2012). The indication for bridging OAC against discontinuation was mainly contributed to the theory that surgical procedures induce hemostatic changes that increase the risk of venous thromboembolism. However, there is yet no evidence that surgery increases the risk of arterial thromboembolism in atrial fibrillation or mechanical heart valves (Kearon and Hirsh 1997). Thus, the risk of discontinuing OAC for a 10-day perioperative period might include a risk for thromboembolism of < 0.18% in patients with atrial fibrillation and < 0.36% in patients with mechanical heart valves (Douketis 2012). In accordance, a large prospective observational study has confirmed the low risk during simple discontinuation of OAC without bridging in minor procedures (Garcia et al. 2008). Furthermore, the bridging OAC-associated risk has been underestimated in the past. The meta-analysis of Siegal et al. demonstrated the increased risk of bleeding complications in particular using full-therapeutic dose LMWH (Siegal et al. 2012).

The first question for surgeons planning an elective procedure in patients on OAC should be: Is the OAC in this particular patient (still) indicated? If not, preoperative discontinuation without bridging clearly seems to be justified. Furthermore, elective procedures might be postponed if only temporary OAC, e.g., after venous thromboembolism, is required. Thus, hernia repair or resection of benign tumors within 3 months after venous thromboembolism requiring an aggressive bridging strategy does not seem to be justified. Secondly, clinicians need to recognize the current individual thromboembolic risk after discontinuation of OAC. This requires simple stratification tools that are available in several guidelines, e.g., the ACCP risk stratification (Hoffmeister et al. 2010; Douketis et al. 2012). Thirdly, clinicians need to question whether the thromboembolic risk clearly outweighs the increased bleeding risk from bridging for the pending surgical procedure. Currently, this question cannot be ultimately answered on an evidence base for every single patient. However, it is clear that most patients do have a low risk of thromboembolism and are likely to benefit from omitting perioperative bridging. Finally, perioperative clinicians need to define the individual bridging strategy including perioperative timing and dosing of LMWH.

**Fig. 1:**
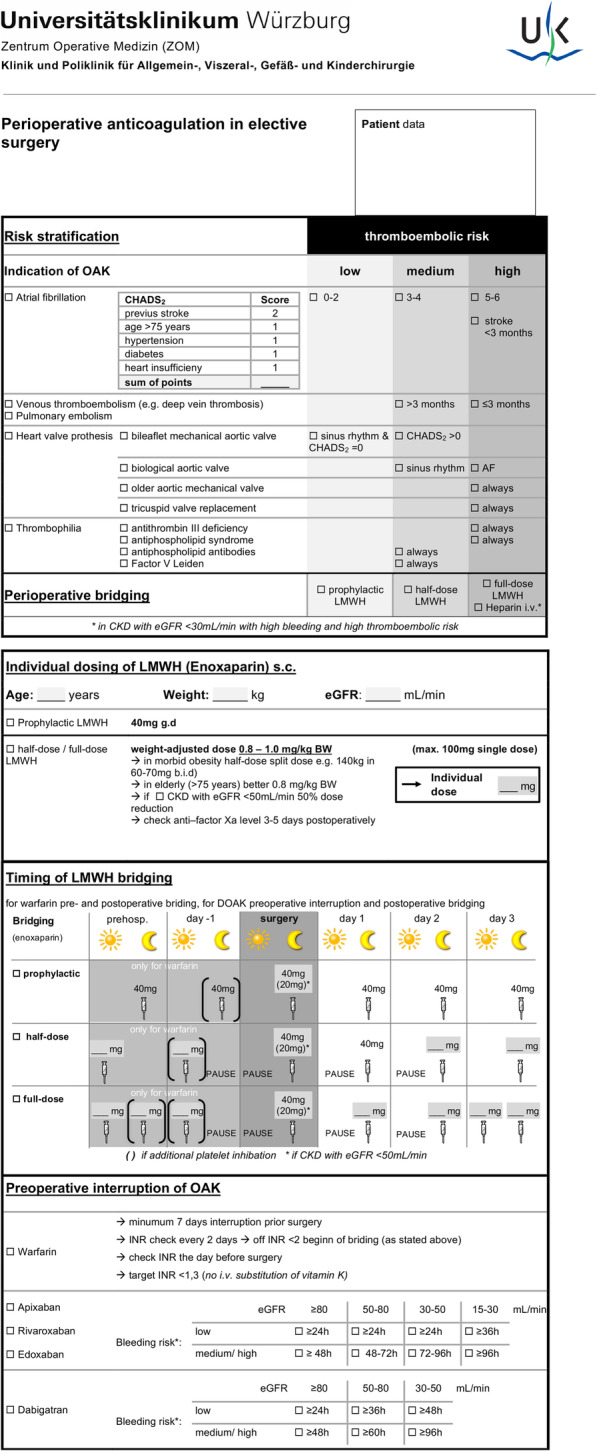
Revised perioperative bridging guideline of our institution

We hypothesize that standardized preoperative risk stratification and an individualized bridging strategy could significantly decrease bridging-associated bleeding risks in general and visceral surgery. Thus, a new multidisciplinary bridging guideline was developed in our hospital (Fig. 1). Further studies are required to confirm its efficacy to reduce bleeding complications while still protecting against thromboembolic events. In addition, future studies should address individual strategies in patients on DOACs and OAC indications with lower prevalence, in particular heart valve replacement and thrombophilia.

## Conclusion

Perioperative bridging anticoagulation, especially full-therapeutic dose LMWH, markedly increases the risk of postoperative bleeding complications in general and visceral surgery. Surgeons should carefully consider the practice of routine bridging for all patients on OAC. A careful balance between the risks of postoperative bleeding complications against the risks of perioperative thromboembolism is mandatory.

## Data Availability

The datasets used and analyzed during the current study are available from the corresponding author on reasonable request.
